# Editorial

**Published:** 1840-04-11

**Authors:** 


### THE MEDICAL EXAMINER.

### PHILADELPHIA, APRIL 11, 1840.

We make some extracts from the eloquent valedictory address of Professor Jackson to the graduates of the University of Pennsylvania. The duty of delivering the address fell unexpectedly upon Dr. Jackson, and it was therefore thrown off nearly with the rapidity of an extempore lecture. This, in our estimation, rather adds to its merits; it gives it the warmth and feeling which are appropriate to a valedictory lecture, instead of smoothing a parting address down to a mere formal farewell. The subject of the address is the duties of a physician in various circumstances, in adding to his professional acquirements and forming a professional character, in contributing to the advancement of medical science, and the duties which he owes to his patients, and to his professional brethren.

The necessity of preparation for opportunities as they may offer, is another prominent division of the lecture. These are numerous enough to occur to all men, and by being ready to fill up the openings that from time to time show themselves, professional progress becomes comparatively easy.

A few days before the delivery of the address, a young physician in whose physiological researches Dr. Jackson had felt a warm interest, ended his short career. A beautiful episode to the memory of the youthful student of nature, finds an appropriate place in the valedictory adddress.

"Human power cannot command and govern the exterior circumstances of the world, and bend them exclusively to man's purposes. They are directed, by a divine and superior agency, to accomplish ends intended from eternity. Men are the instruments made use of for their accomplishment. They are endowed with the qualities fitting them for that object.

"If we cannot change the fixed order of exterior events and circumstances, it is in our power to regulate and control ourselves, to form our principles and characters, to constitute and govern the interior circumstances of our nature. In this manner, man can adapt himself to the events that overtake and involve him. He proceeds with them, may appear to give them direction and control, for he works with them, and reaps fortune and fame: or, should he fall a victim to their overwhelming power when placed in opposition to them, he bows in submission and resignation to the irresistible destiny of a divine law.

"The highest ambition of any individual as it respects this world, should be, to qualify himself by a just understanding and preparation of the powers he possesses, for accomplishing some one of the infinity of ends, that can be perfected in the great movement of our social scheme, by any one generation of men. No one individual, it is probable, more than another, is selected by Divine Providence for a given end. He has provided, in the immense variety of mental, moral and physical qualifications, for the combinations necessary to form the character adapted to any especial purpose. It is always existing. The occasion and the opportunity for the calling of it into action, are alone required, when it appears on the stage, in its place and time.

"The success of one individual more than another, in any particular department of science, or line of pursuit, depends on his being always ready to seize on the opportunity and occasion, as they may offer, by which he can be introduced on the field of action, and his powers be brought into play.

'There is a tide in the affairs of men,
Which, taken in the flood, leads on to fortune.'

"The difficulty that besets most men, is, either that the opportunity does not present itself when they are prepared: or, when it arrives, they have neglected the preparation that is required. Opportunity once lost, is lost for ever. It seldom comes a second time.

"The beautiful apologue of the ten virgins, is not less applicable in a worldly, than in a religious meaning.

"Be like unto the wise virgins, have your lamps trimmed and your oil ready, that when the bridegroom (opportunity) cometh, you may enter in and reap the enjoyment of your foresight and precaution. But if, like the foolish, virgins, you neglect your lamps, have no provision of oil, and when the bridegroom cometh, you have then to look after your neglected means, the door will be shut, and you will in vain seek for admission. Neglect and oblivion will be your portion."

"It is more important that you should obtain, as early as possible, practical knowledge by immediate observation. Neglect no means for this purpose. Frequent hospitals, follow the attendance of dispensaries, bestow your services on the poor, so many of whom require and gladly avail themselves of medical assistance. The principal object you should aim at, is to acquire a knowledge of disease. The symptoms alone should not engage your attention. They are the signs by which a disease is manifested: they are not the disease. What is of still more consequence, is, that you study, by close attention, the natural history of disease, the extent of the natural powers of the economy in their cure, and the methods that nature adopts, in the play of the reactive forces and operations of the system, to disembarrass it of disease. This information is the most certain basis of a safe, sound and judicious treatment. It is to be acquired by the bedside, where you must watch the progress of a case, as it traverses its different stages, and note, in writing, as they occur, the phenomena you witness.

"Most young practitioners mistake the proper object of their clinical studies and observations. They believe the first and great object to be attained, is the prescribing of physic. This is a vulgar notion, cherished by the general ignorance of society as to the true nature of medical science, and the proper character of a physician. It is difficult to resist the importunities of patients and friends of the sick, who expect from the administration of drugs some miraculous influences: it is difficult to divest ourselves of the belief, so flattering to self-love, that with our physic, we are omnipotent in the power of controlling the economy according to our views, and of overcoming disease.

"The last and least important part of the science of medicine is, the dosing of patients with medicines. Understand me: do not suppose I undervalue the immense services derived from the judicious administration of medicines, in the treatment of disease. Medicines produced in the animal economy operations such as nature is observed to excite, as the means of restoration. These processes of nature, the physician imitates; he excites them, artificially, with his medicines, or other remedies. When they are done happily, at the appropriate time, and in accordance with the natural law of the disease, they prove most beneficial, and are curative in their operation. But when the medicinal operation and disturbance are inopportunely provoked, when they come in conflict with the natural law of the disease tending to its solution, confusion and new disorder in the functions and organs are the consequences. The result will be to retard the recovery of the patient, to produce chronic disorders of long suffering, or destroy the power of recovery. *Vel educes quæ educenda non sunt; vel augebis morbum; vel jugulabis ægrotum.*

"Most physicians learn from experience, that often their highest art consists in amusing the patient, inspiring confidence, and thereby quieting the system, that would otherwise be disturbed from nervous agitation, by some imaginary remedies, while nature is permitted undisturbed to accomplish a cure.

"The laws of nature are God's ordinances in the natural world. Man can do nothing without them or against them. It is the first and great object of every scientific practitioner of medicine, to study them and to master them, as they are displayed in the life-mechanism of living beings. Of these laws, it is his pride and boast, that he is the minister and interpreter. He is the servant of God, ministering to and alleviating the temporal and physical wants and infirmities of suffering humanity in the mode of his appointing; just as the ministers of our holy religion are God's servants, ministering to and watching over the spiritual failings and the endangered condition of man's soul, according to his revealed will. Medicine is a mission and a ministry, inferior only to that of religion.

"It is not less an obligation, that you should exert your powers in contributing to advance and improve the science of medicine, than it is to perfect your own knowledge. Medicine, regarded as an art, or a science, all are ready to acknowledge, is imperfect. That it can be advanced to a much higher degree of completeness, cannot be doubted, by any who are familiar with what medicine has been, what has been done within a few years, and what is now doing in the science.

"The advancement of medicine, consists in the greater accuracy and extension of its facts; with an adherence to a more right method of logic and reasoning. It is assuming daily more of the character of a physical and positive, and losing that of a speculative and metaphysical science.

"General theories are but little in vogue. The versatility that prevails in diseases, forbids the expectation, that any one doctrine ever can embrace conditions, so endless, diversified and fluctuating.

"Causes of a general nature, inappreciable except by the phenomena they produce, acting in periodical cycles of varying duration, exert profound modifying influences of different nature, on organized beings, more especially on the vital energies and organization of the human race. From these, result not only the great epidemics, dissimilar at each period, that prevail over whole zones of the globe, but the especial periodical constitutions that impart a common character to nearly all the diseases occurring within that cycle. A doctrine founded on the facts, as they then are observed; and a system of treatment, adapted to a particular constitution, or to a particular epidemic, may be arranged. They will be true for the time: but must fall, as that constitution, or epidemic influence passes away, and a new revolution has brought forward a new train of morbid conditions and phenomena.

"In these circumstances are found the explanation of the diversified theories and modes of practice, that have prevailed at different times in medicine. This has been urged as a reproach on the science and our profession. It is the consequence of things as ordained by the Creator. A theory and practice are true and applicable only for a time. A general and persistent theory is an absurdity in medicine—medical theories must be numerous and variable, for the facts, of which a theory is the aggregate exponent, are themselves, for the most part, complex, variable and transitive.

"You must not, then, wed yourselves to any theory, nor permit yourselves to be enlisted as partizans to any doctrine or practice. Use your theories as a lame man does his crutches; but be ready to throw them aside, as soon as they are useless.

"The advance of medicine, consists in the establishment and verification of facts. But what an endless labour is here opened to the profession. It extends over the whole field of organized beings, vegetable and animal, from the highest to the lowest in the scale, in their natural and diseased, or unnatural conditions. In all these are presented the phenomena of life and organization; the products of life and organization; and the agents that influence the vital and organic phenomena in all their states. These bear with more or less force on medicine, as a science, in illustrating the complicated, obscure, and, without this collateral aid, incomprehensible phenomena of the human economy, the more especial object of medical investigation.

"It is to facts that alone can illustrate medical science, that you should devote your time and attention. Whatever may be the particular bent of your genius, or the kind of talent you possess, there is, in medical researches, some one pursuit adapted to it. You can have no excuse for negligence. The qualifications for these objects, are industry, perseverance, application. These are in the power of each of you. They alone may enable you to establish important truths to be embodied in the science. Facts admitted into science, may be regarded as medallions struck to commemorate an event, or to perpetuate a renown. They carry to remotest time the name of their discoverers.

"The labour given by most men to the acquisition of wealth, applied to scientific objects, would confer on you a celebrity, would make your labours useful to future generations, as to the present. To a generous mind these are far more exciting motives, than the more sordid feelings of avarice."

"Organic phenomena, from their extraordinary complication, could not be approached by analytic processes, until the collateral sciences had reached a sufficient degree of perfection to furnish the means. This period has arrived. Organic phenomena are attacked by every method of analysis. This is exemplified in the history of organic structure. General anatomy, or the reduction of the organs to tissues, commenced in our time, is now completed. The tissues themselves, are now undergoing a further reduction to simpler elements and forms. The microscope, brought to so much perfection, as to be free, to a great extent, from the defects that rendered it at times delusive, is an important means by which this is accomplished. The result is the creation of microscopic anatomy. Two great works are now issuing from the press devoted to this subject. The one, the splendid publication of Professor Berres of Vienna, '*Anatomia Partium Microscopicarum Corporis Humani*:' the other, a more complete and equally splendid work by Professor Mandl, of Strasburg, '*Anatomie Microscopique.*'

"Besides the above large and general works, numerous contributions have been made by other distinguished observers, on the microscopical structure and composition of the tissues and fluids. Professor Henlé of Berlin, has made a most elaborate demonstration of the organization, the physiology and pathology of the mucous tissues. Erdman, Valentine, Burdach, Wagner, and others have furnished new and important facts on the elementary organization of the nerves and muscular tissues.

"Time will not admit of the many examples that could be adduced of the new facts and new views, arising out of them, in anatomy, physiology and pathology, derived from microscopical researches in those departments.

"Organic chemistry is not less rich in its contributions to anatomy, physiology, and pathology, and will soon throw a brilliant light on the darkest spots of our science.

"It will not be accounted rashness, by those who have looked into this subject, when I assert, that under the searching analytical review of the facts of medicine, and application of analytical philosophy to medical science, a large portion of what has been received, and is regarded as established, will be changed, or swept away. Doctrines and opinions founded on those facts, now holding sway, must disappear. They will take their place in the history of the science: they will not belong, as now, to the science.

"But what are we doing in this stirring and busy time, contributing our aid to the improvement of our science? I fear, it must be said, almost nothing: who amongst us is at work in these new fields of scientific research, seeking imperishable fame? I fear, it must be said, no one.

"Three years have this day elapsed, since a young student, full of zeal and ambitious ardor in the pursuit of knowledge, stood on this stage, and received, as you have, the honoured diploma of this school. He presented to the faculty as a thesis, an elaborate essay, in which he confirmed Müller's discovery of lymphatic hearts, or pulsatory lymph organs, in the Batrachia, and extended it by proving their existence in other animals. He did not abandon the course he had commenced so well. He continued cultivating comparative physiology and microscopical investigations, though his means were but moderate. He published as a part of his labours, in the last year, an interesting series of observations on the venous circulation. He was engaged earnestly in pursuing these subjects, and but a short time since, I could have answered the question by adducing Dr. Allison as one, who promised to illustrate by his talents and industry, this department of American science. But, alas, his career is ended. A few days since, and his body was consigned to the tomb. Frail in constitution and delicate in form, he fell a victim to his exertions. A wound, received in dissecting an animal, on which he was making observations, was remotely, as I have been informed, the cause of his death.

"The war-trump, and the muffled drum, and the measured tread of armed men, and the musket shot pealing over the grave, honour the death of the soldier, the slaughterer of his brother man. But the student who meets his death battling for truth in the great arena of science, passes to an unknown grave, followed by the regrets and the tears of the few who knew his worth. Yet there is another judgment, and another reward than that of man. A brighter glory will arise from the obscure grave of the unknown student, than ever yet surrounded the blood-stained monument of the warrior of an hundred fields."

